# Altitudinal population structure and microevolution of the malaria vector *Anopheles cruzii* (Diptera: Culicidae)

**DOI:** 10.1186/s13071-014-0581-8

**Published:** 2014-12-16

**Authors:** Camila Lorenz, Tatiani Cristina Marques, Maria Anice Mureb Sallum, Lincoln Suesdek

**Affiliations:** Instituto Butantan, Avenida Vital Brazil, 1500, São Paulo, CEP 05509-300 Brazil; Biologia da Relação Patógeno-Hospedeiro, Instituto de Ciências Biomédicas, University of Sao Paulo, Av. Prof. Lineu Prestes, 2415, São Paulo, CEP 05508-000 Brazil; Departamento de Epidemiologia, Faculdade de Saúde Pública, Universidade de São Paulo, 1500, São Paulo, CEP 05509-300 Brazil; Instituto de Medicina Tropical, Avenida Dr. Enéas Carvalho de Aguiar 470, São Paulo, CEP 05403-000 Brazil

**Keywords:** Culicidae, Wing, Geometric morphometrics, Mitochondrial gene, Atlantic Forest

## Abstract

**Background:**

In Brazil, the autochthonous transmission of extra-Amazonian malaria occurs mainly in areas of the southeastern coastal Atlantic Forest, where *Anopheles cruzii* is the primary vector. In these locations, the population density of the mosquito varies with altitude (5–263 m above sea level), prompting us to hypothesise that gene flow is also unevenly distributed. Describing the micro-geographical and temporal biological variability of this species may be a key to understanding the dispersion of malaria in the region. We explored the homogeneity of the *An. cruzii* population across its altitudinal range of distribution using wing shape and mtDNA gene analysis. We also assessed the stability of wing geometry over time.

**Methods:**

Larvae were sampled from lowland (5–20 m) and hilltop (81–263 m) areas in a primary Atlantic Forest region, in the municipality of Cananéia (State of São Paulo, Brazil). The right wings of males and females were analysed by standard geometric morphometrics. Eighteen landmarks were digitised for each individual and a discriminant analysis was used to compare samples from the hilltop and lowland. A 400-bp DNA fragment of the mitochondrial cytochrome oxidase gene subunit I (CO-I) was PCR-amplified and sequenced.

**Results:**

Wing shapes were distinct between lowland and hilltop population samples. Results of *cross-validated* tests based on Mahalanobis distances showed that the individuals from both micro-environments were correctly reclassified in a range of 54–96%. The wings of hilltop individuals were larger. The CO-I gene was highly polymorphic (haplotypic diversity = 0.98) and altitudinally structured (Фst = 0.085 and Jaccard = 0.033). We found 60 different haplotypes but only two were shared by the lowland and hilltop populations. Wing shape changed over the brief study period (2009–2013).

**Conclusions:**

Wing geometry and CO-I gene analysis indicated that *An. cruzii* is vertically structured. Wing shape varied rapidly, but altitude structure was maintained. Future investigations should identify the biotic/abiotic causes of these patterns and their implications in the local epidemiology of malaria.

**Electronic supplementary material:**

The online version of this article (doi:10.1186/s13071-014-0581-8) contains supplementary material, which is available to authorized users.

## Background

*Anopheles* (*Kerteszia*) *cruzii* is a neotropical mosquito that employs the bromeliad phytotelmata as a larval habitat. Females blood-feed on humans as well as on other mammals and birds [1]. This mosquito occurs in natural forest ecosystems [2,3] and forest fragments [4]. In spite of its occurrence in forest ecosystems impacted by human activities, *An. cruzii* is mainly a sylvatic species with a low synanthropy index [5]. This mosquito can feed on blood during the day and at night; however, its activity peaks in twilight periods [6-9]. It is a primary vector of *Plasmodium* spp. parasites in areas within the Atlantic Forest biome in Brazil, especially in the States of São Paulo, Paraná, and Santa Catarina [2,10,11].

Individuals of *An. cruzii* were found naturally infected with *Plasmodium* spp. oocysts in the intestine and sporozoites in the salivary glands [12-15]. In rural areas of the Juquitiba and São Vicente municipalities, eastern Sao Paulo state, *An. cruzii* was infected with *Plasmodium vivax* (0.149%), *Plasmodium vivax* strain VK247 (0.086%), and either *Plasmodium brasilianum or Plasmodium malariae* [2]. The occurrence of these species was positively associated with maintenance of transmission of human *Plasmodium* in the Atlantic Forest in the states of Sao Paulo and Rio de Janeiro [14] in cycles that involve humans, *Alouatta*, and *Cebus* primates [11]. The infective biting rate was low in areas in which infected *An. cruzii* were found; however, the abundance of the mosquito and voracious blood-feeding behaviour maintain the endemic circulation of human *Plasmodium* in some areas within the Atlantic Forest domain. Furthermore, *An. cruzii* is a vector of *Plasmodium simium* and *Plasmodium brasilianum*, which cause simian malaria [16,17]. The coastal region of the Atlantic Forest shelters primate species that can be infected by *P. simium* and *P. brasilianum*; these parasites can be occasionally transmitted to humans by infective *Kerteszia* bites [17,18].

In an ecological study conducted in the municipality of Cananéia as biological markers and sampled individuals of *An. cruzii* from in Sao Paulo state, Marques *et al.* [19] found that *An. cruzii* is heterogeneously distributed across different altitudes in the Atlantic Forest. The authors assessed the distribution of *An. cruzii* in three microenvironments, which were grouped based on altitude (lowland: 5–20 m, hill slope: 33–54 m, and hilltop: 81–263 m altitude). Follow-up was monthly for one year; results showed that this species occurred more densely in the hilltop, presumably due to ecological preferences. We then hypothesised that gene flow is not homogeneously distributed across the altitude range and sought to determine the transience of this scenario. The aim of this study was to determine 1) if individuals of *Anopheles cruzii* from lowland and hilltop are morphogenetically similar and 2) if the biological variability of *An. cruzii* is stable over time. To address these questions, we used wing geometry and CO-I mitochondrial DNA sequences as biological markers and sampled individuals of *An. cruzii* from Cananéia municipality from 2009–2013.

## Methods

### Study area

**Figure 1:**
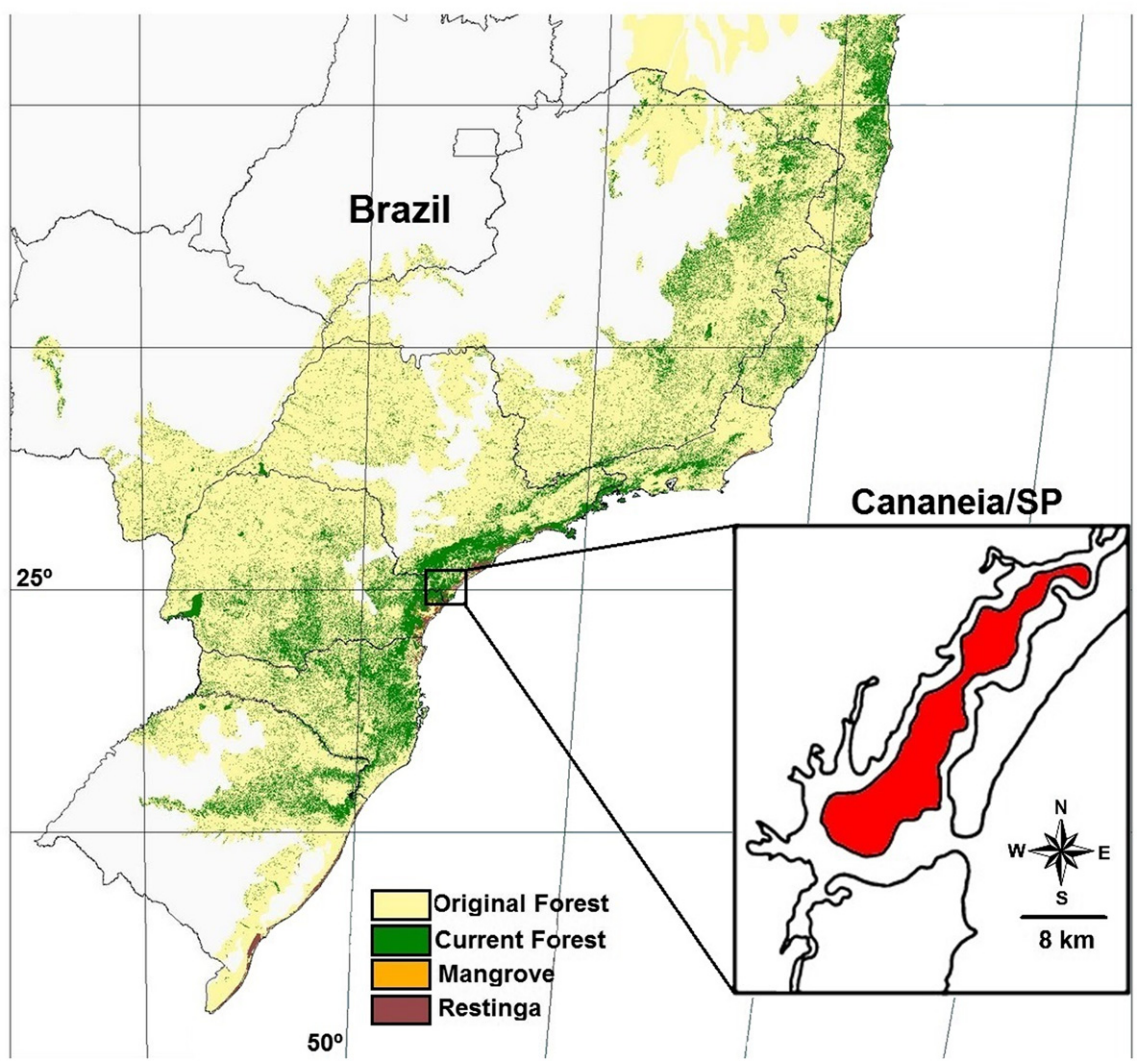
**Study area.** Cananéia municipality in red, modified from IBGE [22].

The municipality of Cananéia is situated in a well-preserved forest in the Atlantic Forest biome (Figure 1), southeastern Sao Paulo state, Brazil. It won status as part of the natural heritage of humanity by the United Nations Organization for Education, Science and Culture (UNESCO) in 1999, because of the ecological importance of its estuarine lagoon complex [20]. According to the Research Centre for Weather and Climate applied to Agriculture [21], the region of Cananéia experiences an annual average temperature of 24°C and average rainfall of 2.8 mm. The vegetation in the area is Submontane Forest that may extend to an altitude of 400 m. Mosquito field collections were conducted in the Aroeira District (25º0’54”S and 47º55’37” W, SAD 69).

### Mosquito collection

Immature *An. cruzii* individuals were taken from water accumulated inside the tanks of terrestrial and epiphytic bromeliads located up to three meters from the ground. Larvae, pupae, and eggs were collected from the water with a manual pump [23] and kept in the laboratory until emergence of the adults. Species identifications were based on characteristics of the fourth-instar larvae and pupae using the identification key proposed by Forattini *et al*. [3] and characteristics suggested by Sallum *et al*. [24].

Collection sites were separated into two microenvironments, lowland (5–20 m altitude) and hilltop (81–263 m altitude) landscape categories, both inside a well preserved forest ecosystem. The lowland areas were characterized by high humidity, high tree density, and little sunlight at ground level. In contrast, hilltop areas were abundant in rocky outcrops, tree density and humidity were low, and there was more sunlight at ground level. Co-ordinates of collection sites are described in Additional file 1. A ground distance of 470 m separates the closest sites between lowland and hilltop; the maximum distance was 1880 m.

Field collections were carried out in January 2012, July 2012, and January 2013. Representatives from the January 2009 population were those reported by Marques *et al*. [19]. The water from each bromeliad tank was kept separate in 500-mL plastic containers in the laboratory under controlled temperature (25 ± 1°C) and humidity (80 ± 10%). Food and larval density in the container were similar for all collections. After emerging, the adults were euthanized and stored in 95% ethanol at -80°C.

### Morphometric analysis

All specimens (Table 1) had their right wings removed and mounted on a microscope slide with Canada balsam. Before mounting, wings were soaked for 12 h in 10% potassium hydroxide (KOH) at room temperature, according to Lorenz *et al*. [25]. Images of the wings were obtained with a Leica DFC320 digital camera coupled to a Leica S6 microscope under 40 × magnifications. All digital images were scored by the principal author (CL).

**Table 1:** **Number of individuals used for geometric morphometrics and date of capture of** ***Anopheles cruzii***

**Month**	**Hilltop**		**Lowland**	
	**♂**	**♀**	**♂**	**♀**
**JAN/2009**	22	26	21	28
**JAN/2012**	22	24	24	26
**JUL/2012**	32	32	26	31
**JAN/2013**	25	25	25	26

**Figure 2:**
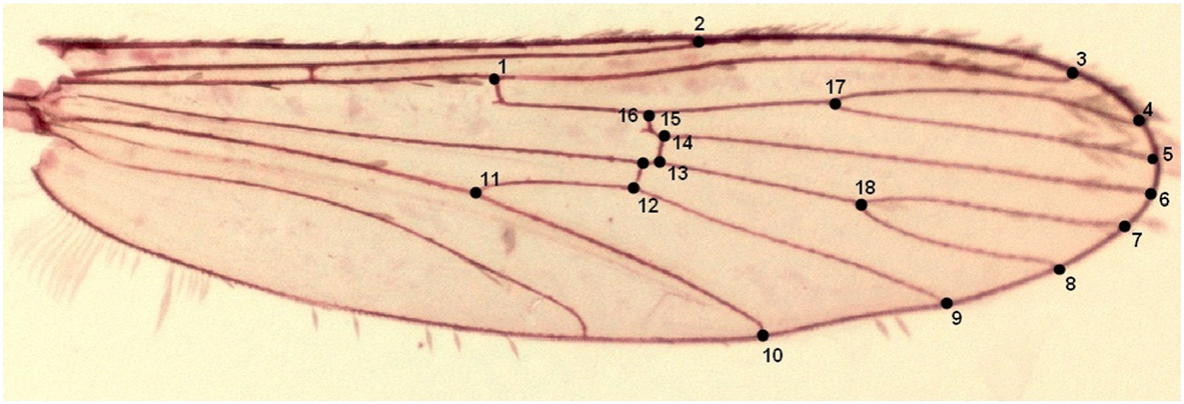
**Wing of** ***Anopheles cruzii.*** Wing dyed with acid fuchsine showing the 18 landmarks chosen for morphometric analysis.

For each wing, the co-ordinates of 18 landmarks previously employed by Lorenz *et al.* [25] and Vidal *et al.* [26] were digitized (Figure 2) and assembled into matrices using TpsDig 2.17 [27]. The co-ordinates were analysed using TpsRelw 1.34 [28] to calculate the consensus configurations and relative warps. The variability in wing shape was assessed using a Canonical Variate Analysis (CVA) to compare populations by landscape and sex. For overall wing sizes of *Anopheles cruzii*, we used the isometric estimator centroid size using TpsRelw 1.34 [28]. The allometric constraints between wing-shape measurements and centroid size were addressed by linear regression of the first relative warp (RW1) on centroid size. To test the accuracy of morphometric classification, each individual was reclassified according to its wing similarity to the average shape of each group (*cross-validation*). The graphics were generated using Statistica 7.0 [29] and edited with Adobe Photoshop 6.01 (Adobe Systems, San Jose, CA) to overlap the 2D splines. Mahalanobis distances were used to estimate metric distance. The Q_ST_ values were estimated from metric variation in wing shape and size according to Dujardin [30]. Q_ST_ separates quantitative genetic variation in a process analogous to Fst for single gene markers [30,31]. Morphometric analyses, Q_ST_, and influential landmarks tests were conducted with COV [32], TET [33], MorphoJ 1.02 [34], and TPS [35].

### Extraction, amplification, and purification of mtDNA

Mosquitoes were homogenized as described by Jowett [36] for extraction of genomic DNA. A total of 96 specimens of *An. cruzii* were used for genetic analysis. We used all the individuals collected in January 2012 (Table 1); the same sample was used for geometric morphometrics analyses. The CO-I gene fragment was PCR amplified in a reaction mixture of 1× buffer (20 mM Tris-HCl, pH = 8.4), 0.4 mM dNTP, 2.5 mM MgCl2, 0.5 mM each primer, 0.5 μl *Taq* polymerase, 2.5 μl genomic DNA, and sterile water to a final volume of 20 μl. A 407-bp fragment of the CO-I gene was amplified with forward primer UEA-7 and reverse primer UEA-10 [37], and did not include the barcode region of mitochondrial DNA. Cycling conditions were as follows: 95°C for 3 min followed by 40 cycles of 94°C for 40 s, 52°C for 40 s, and 72°C for 1 min, and a final extension of 72°C for 10 min. The GenBank accession numbers for the sequences obtained are: KC992738–KC992770. The PCR products were cleaned with a PureLink™ PCR Purification kit (Invitrogen Corporation, Melle, Germany) according to manufacturer instructions. The fragment was sequenced in the forward and reverse directions with ABI PRISM dGTP BigDye® Terminator v3 (Lincoln Centre Drive, Foster City, CA).

### Sequencing mtDNA and data analysis

The purified PCR products and an aliquot of the oligonucleotides specific for the CO-I gene (40 pmol/L) were sent to bio-molecular company Genomic and Molecular Engineering for sequencing on an Applied Biosystems model 3130xl sequencer. These sequences were aligned and edited with the program MEGA 5.0 [38]. The hypothesis of strict neutrality was examined with statistics D [39] and F [40] and was tested with the program DnaSP v5 [41]. Analysis of haplotype diversity and the number of polymorphic sites were calculated with MEGA 5.0 [38] using Kimura 2-parameter distance. The Φst value, which estimates the genetic differentiation by molecular variance (AMOVA), was generated by the software ARLEQUIN 3.5.2.1 [42]. Genetic analysis of population differentiation and nucleotide diversity were also calculated with Arlequin software and a haplotype network was constructed with TCS 1.12 [43]. To test the similarity between hilltop and lowland haplotypes, we used Jaccard’s coefficient according to Real [44].

## Results

### Altitudinal comparisons of chronological samples

**Figure 3:**
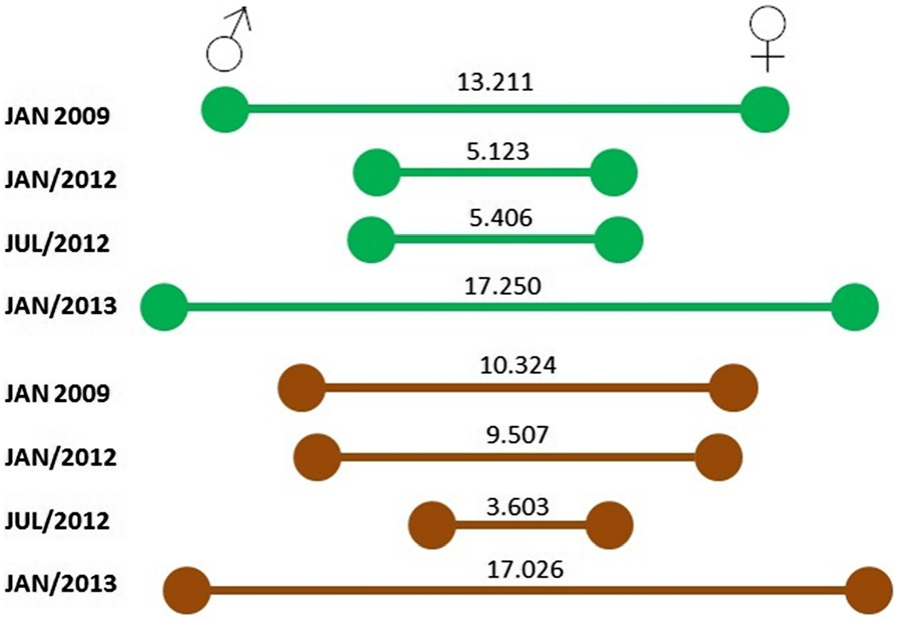
**Sexual dimorphism over time.** Mahalanobis distance between males and females in lowland (green) and hilltop (brown).

Canonical variate analysis revealed sexual dimorphism of wing shape in all populations. To verify that wing shape differentiation was significant between the sexes, the scores of Mahalanobis distance and reclassification between males and females were calculated after removal of the allometric effect of size. The cross-validated reclassification accuracy based on the Mahalanobis distances ranged from 76–100% between males and females (Table 2). The greatest divergence occurred between males and females in lowland samples from January 2013, whereas the least divergence occurred in hilltop samples from July 2012. Figure 3 shows the variation of Mahalanobis distance over time between males and females in the lowland and hilltop populations. Most comparisons showed the wing size of both sexes was similar (data not shown). The most influential landmarks to differentiate males and females in each population were always the same pairs: #10 and #17, #2 and #9, or #9 and #10. With the exception of the population sampled in January 2012, all other collections exhibited significant allometry (Table 2).

**Table 2:** **Data of sexual dimorphism of** ***An. cruzii*** **using geometric morphometrics**

**Month**		**Cross-validation (%)**		**Allometry (%)**	**Influential landmark**
		**♂**	**♀**		
**JAN/2009**	**Hilltop**	81	93	10.470*	#10 and #17
	**Lowland**	100	100	15.968*	#2 and #9
**JAN/2012**	**Hilltop**	87	96	5.853*	#2 and #9
	**Lowland**	76	77	2.141	#10 and #17
**JUL/2012**	**Hilltop**	83	90	6.909*	#2 and #9
	**Lowland**	92	95	5.924*	#9 and #10
**JAN/2013**	**Hilltop**	96	100	8.433*	#9 and #10
	**Lowland**	100	100	3.962*	#9 and #10

(*p < 0.01).

**Figure 4:**
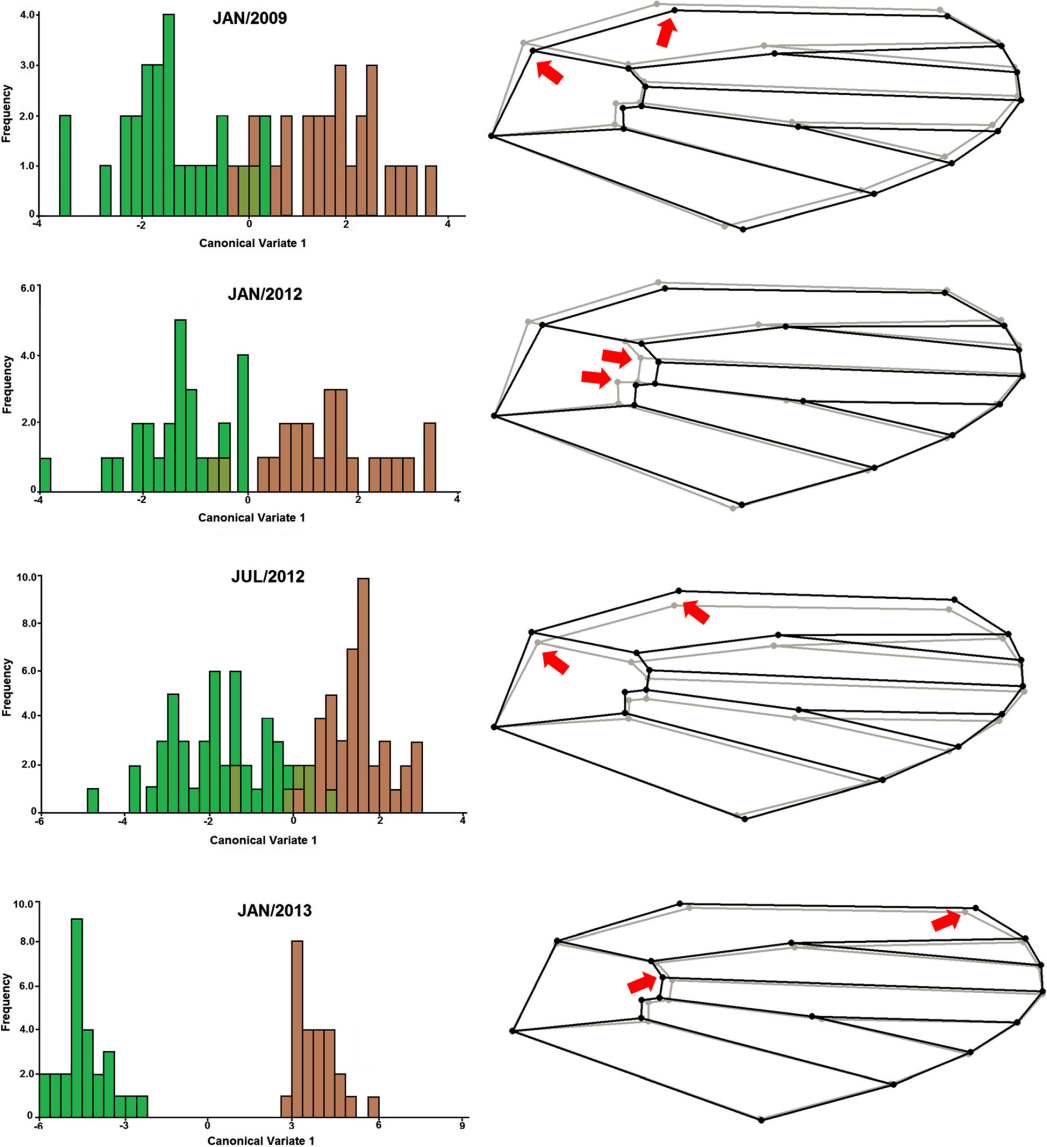
**Geometric morphometrics of females.** Left: morpho-axis of first canonical variable (CV1) originated from the comparison of wing shape between females of lowland (green) and hilltop (brown). Right: wing shape consensus after Procrustes superimposition in lowland (grey) and hilltop (black). Arrows indicate the landmarks of most influence on wing variation.

**Figure 5:**
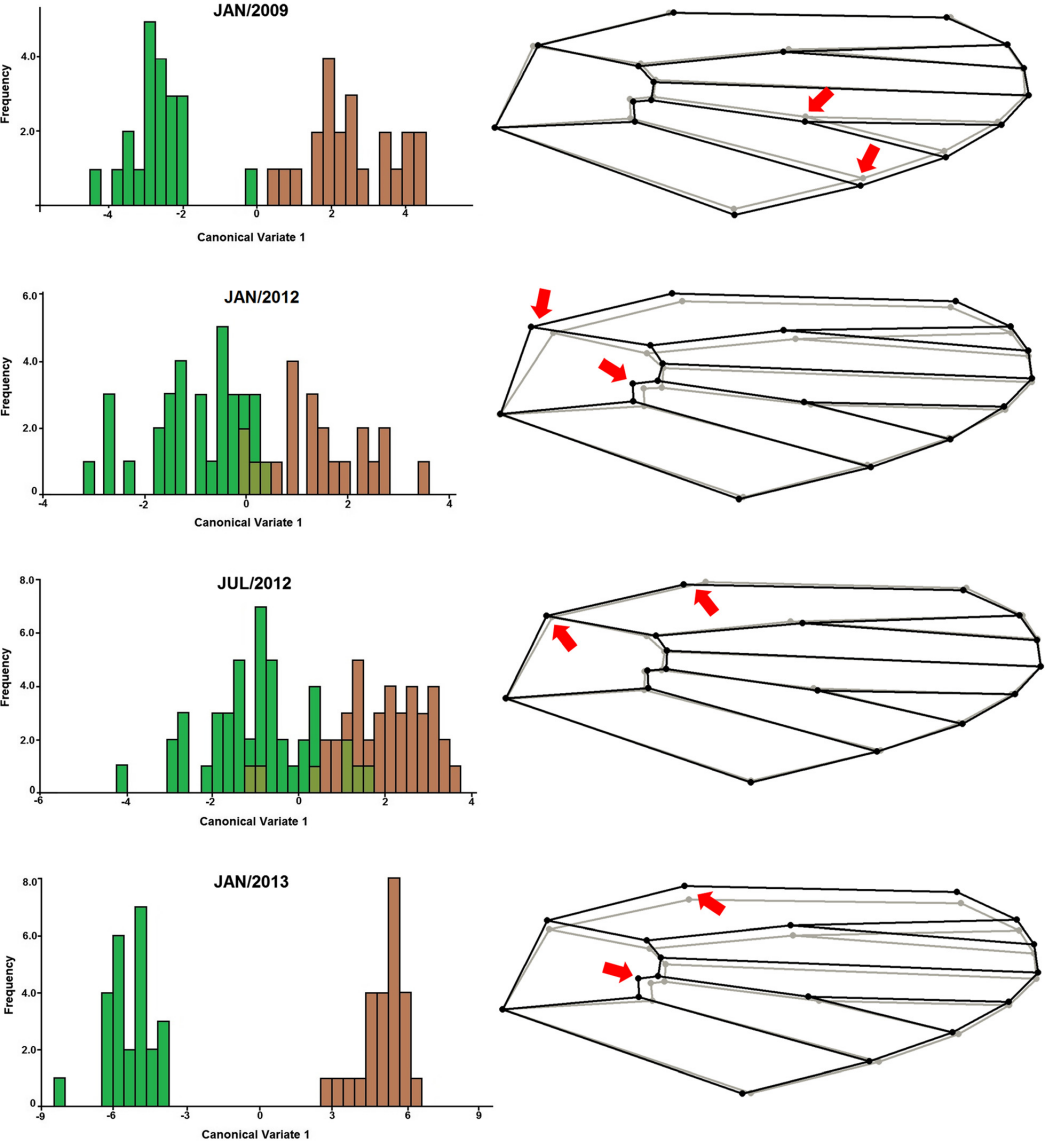
**Geometric morphometrics of males.** Left: morpho-axis of first canonical variable (CV1) originated from the comparison of wing shape between males of lowland (green) and hilltop (brown). Right: wing shape consensus after Procrustes superimposition in lowland (grey) and hilltop (black). Arrows indicate the landmarks of most influence on wing variation.

*Anopheles cruzii* showed sexual dimorphism; thus, we examined males and females separately in the next altitudinal analyses. Comparing the lowland and hilltop microenvironments, we noted that individuals exhibited differentiation of wing shape in all analysed populations. CVA indicated two different morpho-axis groups and we observed a separation between lowland and hilltop specimens (Figures 4 and 5). The comparison of the wing shape consensus after the Procrustes superimposition revealed a different landmark displacement for each microenvironment.

**Figure 6:**
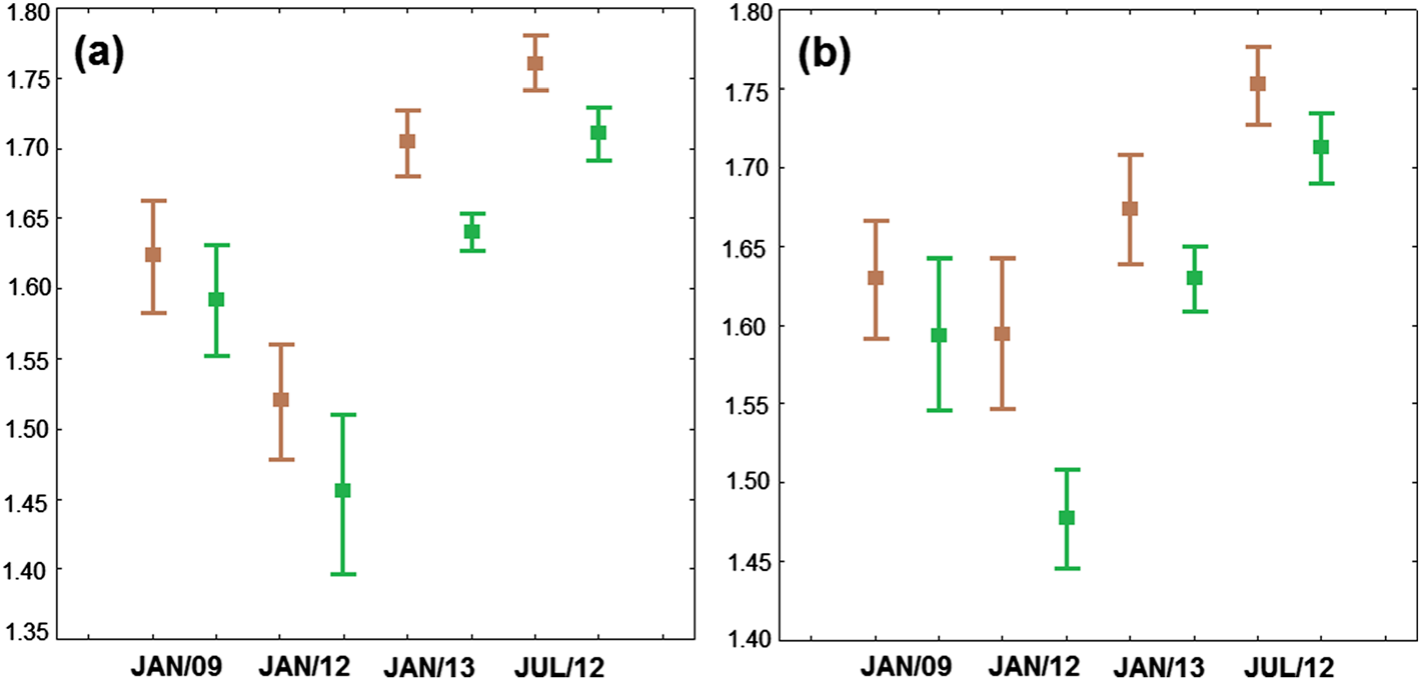
**Centroid size.** Descriptive statistics of wing sizes (in mm) from all populations of sampled *Anopheles cruzii* of lowland (green) and hilltop (brown). **(a)** Females; **(b)** Males.

The cross-validated reclassification accuracy of each individual based on the Mahalanobis distances ranged from 54–96% between lowland and hilltop, according to individual wing similarity to the average shape of each group (Table 3). In terms of allometry, the contribution of size to the shape variation was statistically significant (p < 0.01; both sexes), except in January 2012. The proportion of variance in shape explained by size ranged from 3.46–15.24%, so allometry was removed from the shape analyses. Within each sex, all pairwise size comparisons indicated differences between lowland and hilltop, although some differences were not significant (Table 3). The hilltop mosquitoes were larger than the lowland insects in all populations sampled (Figure 6). The Q_ST_ values for shape were similar in all comparisons and Q_ST_ values for size were considered high only in females sampled in July 2012, January 2013, and in males sampled in January 2012.

**Table 3:** **Altitudinal comparisons between hilltop and lowland using geometric morphometrics**

	**Month**	**Mahalanobis distance**	**Allometry (%)**	**Cross-validation (%)**		**Centroid size (mm)**		**Influential Landmark**	**Q**_**ST**_	
		**Hilltop X Lowland**		**Hilltop**	**Lowland**	**Hilltop**	**Lowland**		**Size**	**Shape**
**♀**	**JAN/2009**	2.980	12.736*	54	63	1.623	1.591	#1 and #2	0.40	0.28
	**JAN/2012**	2.818	3.799	54	53	1.519	1.455	#13 and #15	0.50	0.25
	**JUL/2012**	2.542	6.548*	75	73	1.760*	1.710*	#1 and #2	0.87*	0.27
	**JAN/2013**	8.112	5.359*	91	88	1.704*	1.640*	#3 and #15	0.92*	0.28
**♂**	**JAN/2009**	5.164	15.236*	59	61	1.629	1.593	#9 and #18	0.43	0.25
	**JAN/2012**	2.329	3.462	63	56	1.594*	1.477*	#1 and #13	0.91*	0.27
	**JUL/2012**	2.379	6.018*	69	72	1.752*	1.711*	#1 and #2	0.75	0.31
	**JAN/2013**	10.579	7.935*	88	96	1.673*	1.629*	#2 and #13	0.70	0.22

(*p < 0.01).

### CO-I mitochondrial DNA

All CO-I gene sequences were AT-rich (combined frequency of 71.23%), which is expected within the Insecta [45,46]. There were no non-functional genes (i.e., pseudogenes) as shown by the clear electropherograms, absence of stop codons, and prevalence of synonymous substitutions. A total of 38 substitutions occurred in the mtDNA sequences: 34 were synonymous transitions and four were non-synonymous transversions (Ser > Asn, Leu > Met, Phe > Leu, Met > Leu). The main genetic findings are described in Table 4.

**Table 4:** **Summary statistics for genetic data of** ***Anopheles cruzii*** **: haplotypes, nucleotide diversity and neutrality’s tests**

**Microenvironment**	**H/n**	**Polymorphic sites**	**h**	**π**	**D**_**T**_	***F***
**Hilltop**	33/55	25/407	0.976	0.0087	−1.299	−1.702
**Lowland**	29/41	28/407	0.980	0.0124	−0.867	−1.363
**Hilltop + Lowland**	60/96	38/407	0.986	0.0107	−1.424	−2.156

H = number of haplotypes; n = individuals sampled; h = haplotype diversity; π = nucleotide diversity; D_T_ = Tajima’s D test; *F* = Fu and Li’s F test.

**Figure 7:**
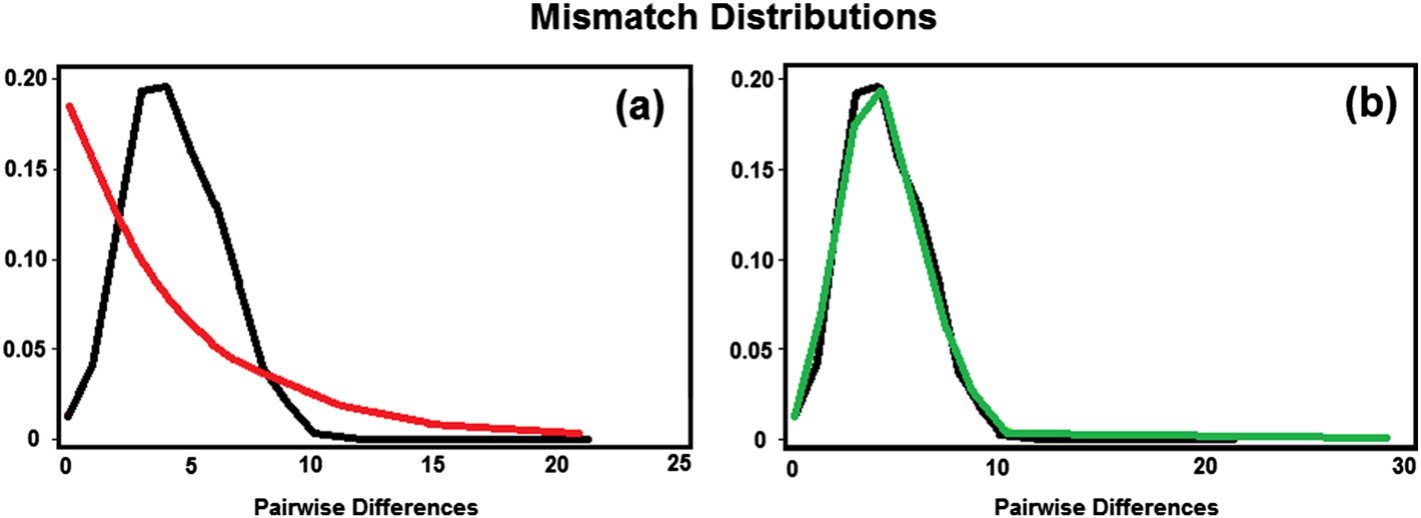
**Population size.** Observed mismatch distributions among haplotypes in populations of lowland and hilltop of *An. cruzii.* **(a)** The red line graph is the expected distribution of a constant size population and **(b)** the green line graph is a growth-decline population. The black line is the observed data.

Haplotypic diversity was high, and the Фst of 0.085 indicates moderate genetic differentiation between the lowland and hilltop populations. The values of Tajima and Fu & Li were not considered significant, accepting the hypothesis of strict neutrality. In spite of this, the graph of mismatch distribution (Figure 7) shows that the populations will probably not have constant size, but are population in growth-decline (p < 0.001).

**Figure 8:**
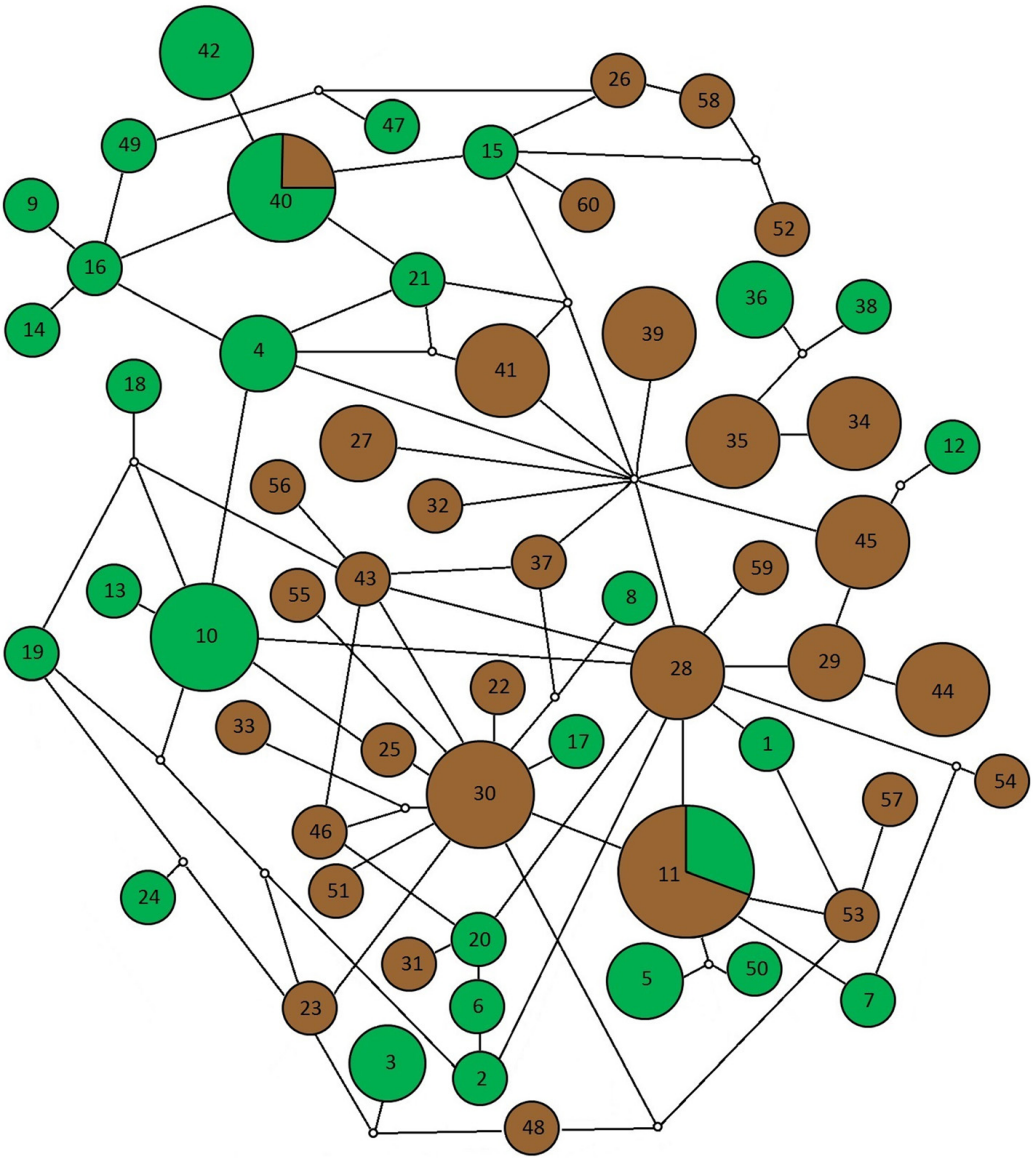
**Parsimony network of the 60 haplotypes.** Circle sizes correspond to the haplotype frequency in lowland (green) and hilltop (brown). The white circles represent a single mutational event. The smallest circles correspond to only one individual.

Among the 96 sampled specimens, we found 60 different haplotypes, only two of which were shared between lowland and hilltop mosquitos (haplotypes #11 and #40). All the exclusive haplotypes were present in very low frequencies; in most cases, only one individual harboured the haplotype. The calculated Jaccard index was 0.033 (p < 0.05), showing low similarity between the two microenvironments. The minimum spanning network illustrates the mutational relationship of the *An. cruzii* haplotypes (Figure 8).

## Discussion

The *An. cruzii* hilltop and lowland populations showed a pattern of wing divergence between all sampled sets. The minimum ground distance between these micro-regions is approximately 470 m, so the mosquitoes could fly between them [47]. Apparently, there are other ecological reasons that prevent them from freely circulating between altitudes. The preference for specific biotopes and bromeliads within each microenvironment may differ in each population. The micro-environmental context may influence behavior, genetic drift, natural selection and indirectly drive genotype and phenotype. This hypothesis was also discussed in a study case conducted in the same locality: Marques *et al.* [19] found that the volume of water in lowland bromeliads is significantly higher than in hilltop plants; this could be a determining factor in the preferences of each population. The landscapes of hilltop and lowland also differ in other respects: the levels of sunlight, vegetation cover, relative humidity, and resident plant species may influence the preference of anopheline populations and, thus, their differentiation.

Veloso [48] observed that the species is indifferent to special microclimates, being found in large quantity in various types of biotopes. If the individuals were under selective pressure, as has already been observed in other species of culicids, there would be a tendency to reduce the intraspecific variability of this characteristic [49]; this was not observed in our study. However, the separation of groups by wing shape indicates a divergence within the species *An. cruzii.* Micro-environmental variation might affect wing shape, and this interference can be related to flight performance in Culicidae [50]. In *Drosophila*, numerous genes control natural variation in wing shape [51], and the development of shape requires a cascade of genes that act throughout development; this may explain why changes in wing shape are sensitive to a variety of environmental stressors [52] and that minor perturbations during development can lead to large changes in shape [53].

Although wing shape varied over time, altitudinal structure and sexual wing dimorphism were maintained. Despite its dynamic and rich variability, wings are evolutionarily informative and appear to be canalized. The morphological variation differential for each sex can be the result of different selective pressures that can shape the wings as part of their adaptations. Models by Lande [54] and Lande and Arnold [55] assume that the genetic basis for sexual dimorphism is polygenic. Males and females have different ecological roles in the environment and use their wings differently; this could motivate sex-specific natural selection [56]. The comparison of wing shape consensus after Procrustes superimposition revealed that the most influential landmark in the majority of populations was landmark #9, localized in the wing border. It seems that this anatomical mark is important in sexual dimorphism of this species. According to Dujardin [30], the landmarks are differentially affected by the same displacement. The posterior border of the wing moved in opposite ways, depending on the external stimulus [30]. In terms of allometry, the contribution of size to the shape variation was statistically significant for most populations; however, the elimination of allometry before CVA allowed us to say that the observed variation probably is not due to plasticity.

In this study, we found a very high number of haplotypes for each microenvironment sampled, only two of which were shared between lowland and hilltop insects. The nucleotide diversity was also high, indicating that the analysed fragment of CO-I is extremely polymorphic in *An. cruzii*. The large number of haplotypes found differs from other studies of the anopheline CO-I gene [57,58]. The high frequency of transitions in the third codon position indicates that the analysed groups diverged recently [59]. There have been no published population-based studies of mtDNA in *An. cruzii* [60]. The large quantity of haplotypes found in micro-environmental comparisons may reflect the proximity to the centre of origin of this species. The distribution of *An. cruzii* is restricted to South America [3]; thus, its centre of origin is also located in this region. In general, older populations have a higher diversity than younger populations [61,62].

Comparing the values of genetic diversity (Φ_ST_) with those of morphological diversity (Qst), we noted that Qst of shape and size, were higher. This reveals a greater degree of evolution in the wing than in the CO-I gene; therefore, these markers evolve at different rates. If the markers were neutral they should converge to the same value [30], which is not the case here. In *An. cruzii*, the CO-I gene was so polymorphic that might not be the most appropriate marker to demonstrate a clear pattern of divergence between populations, as in *Anopheles darlingi* [63,64] or *Aedes aegypti* [65]. Although Tajima [39] and Fu and Li’s [40] F neutrality tests did not observe significant negative population values (Table 4), the results of mismatch distribution analyses indicated that the population probably did not have a constant size, but remained in growth-decline (Figure 7).

Studies of *Culex* [66] and *Aedes* [67] have demonstrated that altitudinal stratification can be revealed by individual wing morphology; however those studies have compared distant populations, with altitudes ranging from 800 to 2130 m. Our study revealed variation between closely located populations, where the geographical distance between microenvironments was not substantial (see Additional file 1). An incidental hypothesis of our work is that the lowland/hilltop populations are undergoing an incipient speciation process. The presence of cryptic species under the nominal species *An. cruzii* has been reported in other localities, but the occurrence of this phenomenon in Cananéia is still controversial [68,69]. To test this hypothesis, new studies should be performed using the other taxonomical markers traditionally employed to diagnose cryptic species in *An. cruzii*: chromosomes [68,70,71].

## Conclusions

The data for wing shape and gene CO-I were concordant, which probably indicates the vertical structure of *An. cruzii* in the Cananéia region. Despite microevolution over time, the altitudinal structure and sexual wing dimorphism were maintained in this species. The hilltop and lowland populations differ in several aspects, which might be also reflected in their vectorial capacity. Our proposed scenario relies on theoretical conditions conducive to development of parapatric speciation, in which there is no specific extrinsic barrier to gene flow. The population is continuous, but the individuals do not cross randomly [72]. They are more prone to cross with their geographical neighbours than with individuals of a different group. There is continuity between the landscape microenvironments, because the bromeliaceous breeding sites are present throughout the forest and there are no physical barriers between lowland and hilltop. However, the morphological and molecular evidence suggests that the anophelines do not pass randomly through the vertical stratum. Knowledge of these phenomena will support our understanding of the transmission dynamics of the *Plasmodium sp*. It is also possible that the gene flow patterns differ throughout the year or that population makes demographic substitution. The gene variability in populations is unknown; however, if this scenario changes, it can be used to understand the epidemiology of malaria in the region. It opens a demand for studies on sexual dimorphism and genetic basis of altitudinal preference.

## Additional file

Additional file 1:
**Map and geographic coordinates of collection sites.**
