# Supplementary material for: Altitudinal population structure and microevolution of the malaria vector Anopheles cruzii (Diptera: Culicidae)
Source: Parasit Vectors. 2014 Dec 16;7:581. doi: 10.1186/s13071-014-0581-8 (PMC4334843; doi:10.1186/s13071-014-0581-8)
Supplement: Additional file 1: — Map and geographic coordinates of collection sites. [file 13071_2014_581_MOESM1_ESM.pdf]

**Map and geographic coordinates of collection sites**

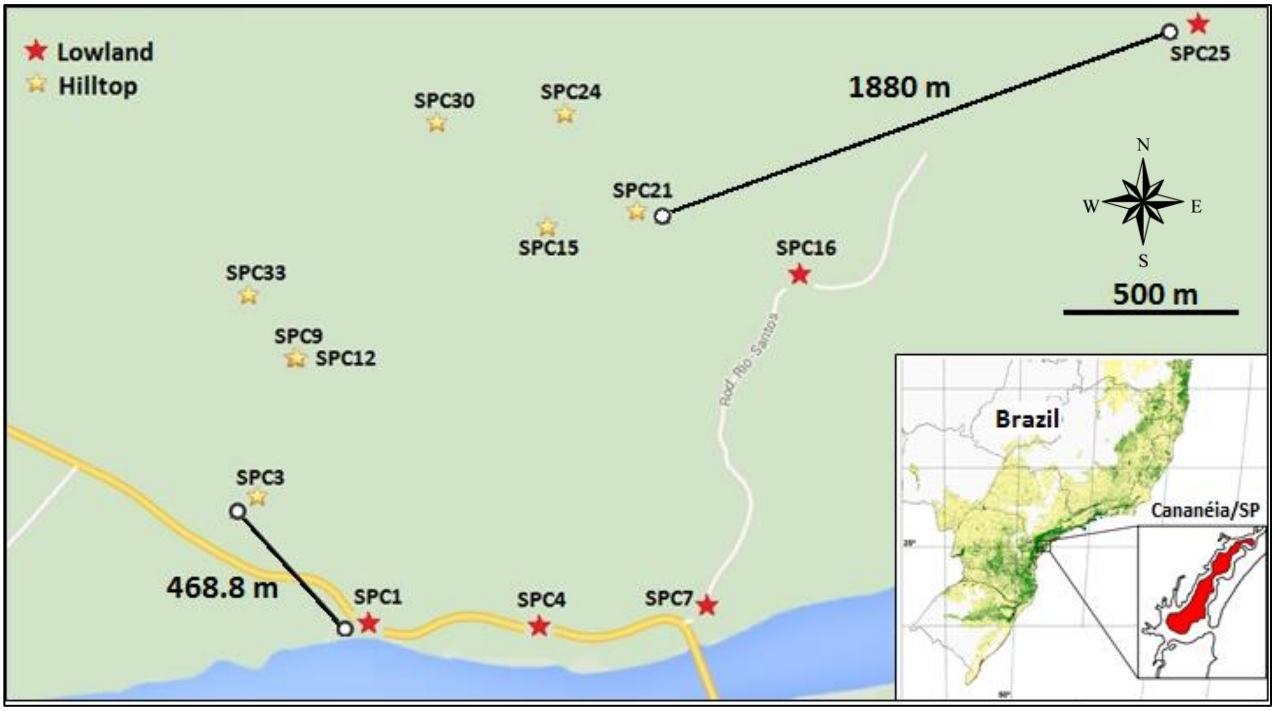


Collection points of Cananéia/SP. The minimum distance between lowland and hilltop is approximately 470 m (*Google Maps*)

Coordinates of the collection points:

**Lowland sampling sites:**

SPC1 –24°53'26.8"S 47°50'52.1"W
SPC4 - 24°53'27.2"S 47°50'33.9"W
SPC7 - 24°53'25.2"S 47°50'16.1"W
SPC16 - 24°52'53.7"S 47°50'06.4"W
SPC25 - 24°52'29.8"S 47°49'24.8"W

**Hilltop sampling sites:**

SPC3 - 24°53'14.8"S 47°51'03.4"W
SPC9 - 24°53'01.6"S 47°50'59.5"W
SPC12 - 24°53'01.6"S 47°50'59.3"W
SPC15 - 24°52'55.6"S 47°51'04.3"W
SPC21 - 24°52'38.4"S 47°50'31.1"W
SPC24 - 24°52'39.2"S 47°50'44.5"W
SPC30 - 24°52'47.6"S 47°50'23.5"W
SPC33 - 24°52'49.1"S 47°50'32.9"W
